# Oncoviruses Can Drive Cancer by Rewiring Signaling Pathways Through Interface Mimicry

**DOI:** 10.3389/fonc.2019.01236

**Published:** 2019-11-15

**Authors:** Emine Guven-Maiorov, Chung-Jung Tsai, Ruth Nussinov

**Affiliations:** ^1^Computational Structural Biology Section, Basic Science Program, Frederick National Laboratory for Cancer Research, Frederick, MD, United States; ^2^Department of Human Genetics and Molecular Medicine, Sackler Institute of Molecular Medicine, Sackler School of Medicine, Tel Aviv University, Tel Aviv, Israel

**Keywords:** pathogen-driven cancer, host-microbe interaction, host-pathogen interaction, interface mimicry, computational prediction/modeling, protein-protein interaction, structure, superorganism network

## Abstract

Oncoviruses rewire host pathways to subvert host immunity and promote their survival and proliferation. However, exactly how is challenging to understand. Here, by employing the first and to date only interface-based host-microbe interaction (HMI) prediction method, we explore a pivotal strategy oncoviruses use to drive cancer: mimicking binding surfaces—interfaces—of human proteins. We show that oncoviruses can target key human network proteins and transform cells by acquisition of cancer hallmarks. Experimental large-scale mapping of HMIs is difficult and individual HMIs do not permit in-depth grasp of tumorigenic virulence mechanisms. Our computational approach is tractable and 3D structural HMI models can help elucidate pathogenesis mechanisms and facilitate drug design. We observe that many host proteins are unique targets for certain oncoviruses, whereas others are common to several, suggesting similar infectious strategies. A rough estimation of our false discovery rate based on the tissue expression of oncovirus-targeted human proteins is 25%.

## Introduction

About 15–20% of all human cancer incidents have viral etiology (1–3), with evidence mounting for the carcinogenicity of several viruses (4). Cancer-causing viruses, also known as oncoviruses, include human papilloma virus (HPV) which causes cancer of cervix, vulva, vagina, penis, anus, and head and neck; Kaposi's sarcoma herpes virus (KSHV) which causes Kaposi sarcoma and primary effusion lymphoma; human T-lymphotropic virus 1 (HTLV1) which causes adult T-cell leukemia/lymphoma; Epstein Barr virus (EBV) which causes Burkitt lymphoma, immunosuppression-related non-Hodgkin lymphoma, extranodal NK/T-cell lymphoma (nasal type), Hodgkin lymphoma, and cancer of the nasopharynx; Hepatitis C virus (HCV), whose chronic infection causes hepatocellular carcinoma and non-Hodgkin lymphoma; Hepatitis B virus (HBV), whose chronic infection causes hepatocellular carcinoma (4); Merkel cell polyomavirus (MCPyV) (5, 6); and human cytomegalovirus (HCMV) (7). Although the oncogenic roles of HCMV and MCPyV are still debated, we included them here to have a more comprehensive study. Even though they belong to diverse viral families, with DNA or RNA genomes and varied oncogenic mechanisms, they share some common features: (i) Their infections are seen in many, but most infected individuals do not develop cancer. (ii) They do not lyse the host cell, instead persist latently for a long time. This immune evasion strategy allows viruses to hide from host immunity. A long latent phase in their biological cycle does not exclude their potential to enter the lytic cycle. (iii) Despite their causative roles in cancer, in general they are insufficient to trigger tumorigenesis. They require additional risk factors, such as immune suppression, chronic inflammation, co-infection with other pathogens, and host mutations (8). KSHV and HPV are considered necessary in Kaposi's sarcoma and cervical cancer, respectively, since they are always present in these tumors. Tumorigenesis is not the goal of these viruses, rather an “unfortunate consequence” of their infection and survival capabilities. Oncoviruses are classified into direct and indirect carcinogens. Direct tumor viruses [HPV, KSHV, HTLV1, EBV (4), and MCPyV (6)] either encode viral oncoproteins or activate host oncoproteins. Indirect viruses (HBV and HCV) however, set the stage for neoplasm mainly by chronic inflammation. Despite having a viral oncoprotein HBx which cooperates with cellular oncoprotein RMP, HBV is classified as an indirect carcinogen (9). A broad range of infectious agents cause chronic inflammation that are not associated with cancer. Some indirect viruses set the stage for cancer by immune suppression, such as HIV-1 (4).

Despite considerable data on their contribution to cancer, the exact molecular mechanisms of how they reprogram the host pathways to elicit malignant transformation remains unclear. Carcinogenesis is a multistep process and oncoviruses can exert their effects at any step (10). The presence of viral oncoproteins, such as HPV E6 and E7 proteins, induction of host oncoproteins by viruses, inactivation of host tumor suppressors by viral proteins, and altered host gene expression due to viral genome integration into the host genome are the main causes in virus-driven oncogenesis. Protein-protein interactions between the host and the virus, below referred to as “host-microbe interactions (HMIs),” play important roles in rewiring host pathways and as such have significant roles in tumor initiation or progression in virus-associated tumors. With small genome sizes, viruses encode only a few proteins, even though there are some exceptions, such as HBV genome which encodes more than 85 proteins. Still, compared to genomes of other pathogens, like bacteria, they encode a small number. Except for the established oncoproteins, it is still unclear whether additional viral proteins play a role in the malignant conversion of the host cell. Although they may not have direct oncogenic effects, they may be essential in virus-induced tumorigenic processes, exerting pleiotropic effects during initiation or maintenance of the malignant phenotype. The impact of individual viral proteins in reprogramming the host interactome appears proportional to the number of their HMIs (11). To modulate host signaling with only relatively few proteins, viruses target regulatory nodes in the host (12, 13). These nodes are also subject to mutations in non-virus-induced cancers (5). Thus, large-scale detection of HMIs and their structures can help delineate the functions of viral proteins.

**Figure 1:**
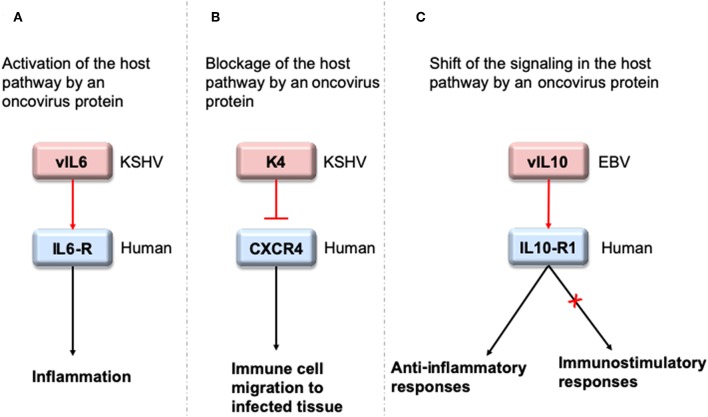
HMIs can activate, inhibit or shift host signaling pathways. **(A)** KSHV cytokine mimic vIL6 serves as an agonist to host IL6-R and initiates host inflammatory responses (21). **(B)** KSHV chemokine mimic K4 antagonizes cellular chemokine receptor CXCR4 and inhibits immune cell recruitment to the infected tissue/cell (22). **(C)** EBV cytokine mimic vIL10 binds to cellular IL10-R1 with a ~1,000 fold less affinity compared to its host counterpart. While it activates the host anti-inflammatory responses, it cannot activate other immunostimulatory functions, such as stimulation of thymocyte and mast cell proliferation (23). Pink and blue proteins are from virus and human, respectively. Red arrows indicate oncovirus action/impact on a host protein and black arrows are the conventional downstream outcome of the host pathways.

To interact with host proteins and subvert their signaling, one of the strategies microbial proteins use is molecular mimicry where they mimic interactions of the host (14). Molecular mimicry occurs in four different ways: hijacking (i) full-length protein sequence, (ii) short sequence, also known as “motif mimicry,” (iii) global structure even with limited sequence similarity, and (iv) structure of a binding surface, so-called “interface mimicry.” Interface mimicry seems much more common than global sequence and structural similarity. Interfaces are favorable scaffolds that are re-used by proteins with distinct global structures to bind to their partners (15–18). Interface mimicry can be seen in endogenous (intra-species) (19) and exogenous (across-species) interactions (12, 20). Such mimicry allows pathogenic proteins to compete with their host counterparts and interfere with the host endogenous protein-protein interactions (PPIs). By hijacking only one host interface, which is utilized by several other host proteins, microbes may affect several host PPIs simultaneously. Microbe proteins can activate, block, or shift host signaling (Figure 1).

**Figure 2:**
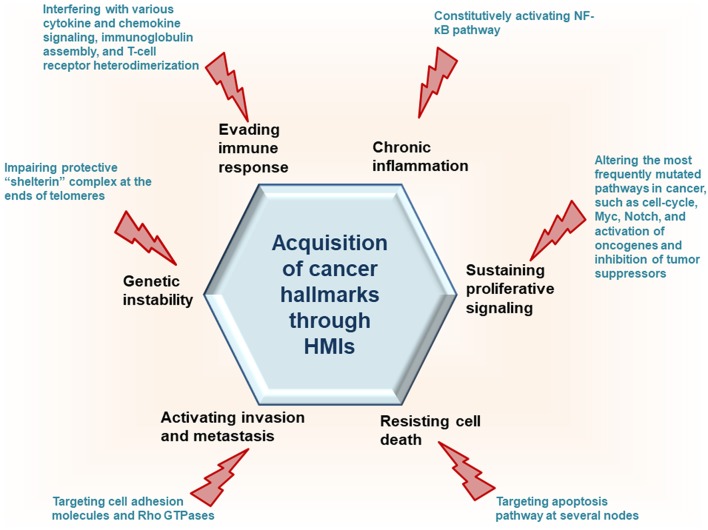
HMIs can prompt host cells to attain cancer hallmark traits.

Recently, we developed a powerful interface-based HMI prediction method—HMI-PRED—, which can be applied to pathogens or commensals on a proteome-wide scale [(24, 25), Guven-Maiorov et al., under revision]. HMI-PRED is complementary to experimental methods and can predict many more HMIs than currently detected by experiments. Here, using this method, we modeled the HMIs of all oncoviral proteins with available 3D structures, deciphering the molecular basis of how they may facilitate acquisition of cancer hallmarks (Figure 2). We identified 6,034 potential HMIs for 51 proteins of 8 known oncoviruses. We further found that oncoviruses target several key pathways in cancer, such as cell cycle, PI3K, RTK-Ras, and MYC. Importantly, 202 of the virus-targeted host proteins are oncogenes and tumor suppressors, indicating that oncoviruses exploit the same proteins and pathways that non-virus induced cancers do. To the best of our knowledge, this is the first study that enriches the structural HMI space of all known oncoviruses, constructs the superorganism structural network and sheds light on possible oncoviral transformation strategies through such interactions. Without the structures of HMI complexes and the host PPIs that they affect, the list of only potential viral targets in the host would not be sufficient to comprehend the molecular basis of viral contribution to malignant transformation.

## Methods

### Modeling HMIs

Here, to model the oncoviral HMIs and uncover their complex (bound) 3D structures, we employed a user friendly webserver (HMI-PRED, http://interactome.ku.edu.tr/hmi), which utilizes the first and to date only interface-based HMI prediction method that we developed recently [(24, 25), Guven-Maiorov et al., under revision]. The rationale behind our method is that exogenous interfaces mimic endogenous ones to interact with host proteins. The main function of docking tools is modeling the complex structures of proteins that are known to bind to each other. However, in HMI prediction, the main aim is to detect the interacting partners of pathogenic proteins in the host. With our method, we can identify not only the interacting HMI partners, but also their bound structures. The only input for our method is the structure of microbial protein. The input microbial structure needs to have more than 15 residues to model the HMI because shorter peptides cannot meet the “match thresholds” for the interface alignment, which is at least 15 residues and 1 hotspot residue should be aligned with the template interface.

To build our template interface set, we extract all human interfaces—both endogenous and exogenous—from all available human protein structures in Protein Databank, deposited as of January 2019, as described in (26). We clustered the redundant interfaces corresponding to same human protein interaction and select a representative for each cluster. The non-redundant template interface set has 17,351 human interfaces, corresponding to 3,555 human PPIs and 691 human exogenous interactions with other microbes. Every interface has 2 faces, 1 from each interacting partner in the host PPI. At least 1 face of the interface is human protein.

In the first step of our approach, we structurally align the pathogenic protein with the template interfaces to determine the potential HMI pairs. The interface matching thresholds are the same as that of PRISM (27–30): at least 15 residues and 1 hotspot residue of the template interface should match with the microbial protein. We perform structural alignment by either TM-align (default) or Multiprot. In this study, we used TM-align, with a threshold of 0.25 TM-score, which ranges from 0 to 1 (For more stringent alignment, the user can set a higher threshold). If the microbe protein is aligned with the first face of the interface, it can interact with the complementary second face. These putative HMI pairs have structural complementarity, which does not necessarily confer electrochemical complementarity, i.e., favorable interaction energy. To determine the energetically favorable HMI pairs, we utilize Rosetta (local refinement) (31). We regard HMIs as energetically favorable, only if their Rosetta interface scores (I_sc) are smaller than −5 and total energy scores smaller than zero. We also calculate the I_sc of template interfaces (endogenous human PPIs) and compare them with that of the HMI models to see whether these putative HMIs will outcompete their human counterparts. To further assess the probability of the HMI models to be real HMIs and decrease our error rates, we calculate the percent-match (ratio of the number of aligned residues to the number of template interface residues). We assign a weight to each template interface such that bigger interfaces have larger weights. If the template interface has <30 residues (*n* < 30), the weight is 0.5; if 30 < *n* < 50, weight is 1; if 50 < *n* < 80 weight is 1.5; and if *n* > 80, the weight is 2. Lastly, we calculate the probability of template interfaces being real biological interfaces, instead of crystal artifacts, with the EPPIC (Evolutionary Protein-Protein Interface Classifier) (32). Score 3 given in Tables S1, S2 incorporates the I_sc, percent match, assigned weights and the probability score that the EPPIC server gives. The lower the Score 3 is, the higher chances of the HMI models to occur since they hijack the real biological interfaces better.

### Rough Estimation of False Discovery Rate

Due to scarcity of experimentally available HMI data, it is hard to calculate the exact false discovery rate of our predictions. We estimated the false discovery rates based on tissue expression of the human proteins, by considering oncovirus-targeted host proteins that are known to not be expressed in the oncovirus-infected host tissue as false-positives. Theoretically they can interact with the oncoviral proteins, but if they are not expressed in the tissue(s) where the oncovirus is found, the HMIs through these human proteins cannot take place. The tissue expression data is obtained from Human Protein Atlas (33) and the details of the tissue expression information for each oncovirus are given in Table S3. The average false discovery rate of our predictions for eight oncoviruses is 25.47%. Importantly, the HMIs that can occur in the infected host tissue—according to the tissue expression data—may also have false positives, but we cannot calculate it due to limited experimental data.

### Statistical Analysis of the Enrichment of Oncogene/Tumor Suppressor Proteins in Oncovirus-Targeted Host Proteins

We performed a Chi square test and found that the enrichment of oncovirus-targeted host proteins in oncogenes and tumor suppressors is statistically significant (chi^2^ = 98.32, *p* = 3.54e-23, df = 1).

We found 6,034 HMIs for 51 oncoviral proteins. There are 2,448 distinct human proteins in these 6,034 HMIs, 202 of which are known human oncogenes and tumor suppressors according to COSMIC Cancer Gene Census (release v85, 8th May 2018). In our template set, there are 17,351 human interfaces (human PPIs) and 4,762 distinct human proteins in these PPIs. Two hundred and forty-five of these 4,762 human proteins are known oncogenes and tumor suppressors. We calculated the *p*-value with “chi2_contingency” function in “scipy.stats” library of python.

### Constructing the Structural Superorganism Network

Since we have bound structures of both modeled HMIs and endogenous human PPIs (template set), we can build the structural superorganism interaction network. We have 6,034 non-redundant HMIs and 6,456 human protein interactions in the network. We visualized the network with Cytoscape (34) and calculated its topological features with NetworkAnalyzer (35). Functional annotation and the enriched KEGG pathways of the human proteins that are targeted by the oncoviruses were performed by DAVID (36, 37).

## Results

We analyzed 51 viral proteins from 8 oncoviruses with our interface-based HMI prediction approach (24, 25), obtaining 6,988 candidate HMIs. Details of the HMI models and the endogenous human PPIs that they may disrupt are given in Table S1. Our analysis included all oncoviral proteins with available 3D structures in the PDB, covering at least 15 residues, regardless of whether they are viral oncoproteins. Some HMIs appear more than once in the table, because they have different modes of binding with the same host protein (i.e., they are identified through distinct template interfaces). There are 6,034 non-redundant HMIs, excluding different binding modes with the same host target. In addition to endogenous host interfaces, viral proteins can also mimic exogenous interfaces of other pathogens with host proteins (Table S2). For example, Flice inhibitory protein of KSHV (vFLIP_KSHV) may interact with human P53 (P53_HUMAN) since it has very similar interface to Large T antigen protein of simian virus 40 (LT_SV40). Hijacking other exogenous interfaces may allow GP350_EBV, E7_HPV, and vBCL2_KSHV to bind to pro-apoptotic protein BAK_HUMAN, suggesting convergent evolution of these viruses ending up with similar infectious strategies. Although some viral proteins are known to function as oncoproteins, they may have weak activity, such as Tax_HTLV1 (10), suggesting possible involvement of other viral proteins in transforming the host cell. Rough estimation of the average false discovery rate of our predictions for 8 oncoviruses, based on tissue expression is 25% (see Methods for details). Below, we describe the recovery of known HMIs, provide examples of the novel predictions and how they can elucidate oncoviral transforming strategies, describe common targets of oncoviruses, and present the structural superorganism network.

### Recovery of Known HMIs

Although some viral proteins have experimentally identified HMI data, for others, such as BMRF1_EBV, GP350_EBV, and NEC1_HCMV, there are no known interactors in the host. Most of the HMIs that we found are novel, but we also recovered some that are known. We enrich the oncoviral HMI data and provide the structures of their complexes. Table S4 lists the structurally known oncovirus interactions with host proteins. Only 25 known interactions of oncoviruses have resolved structures. We recovered 17 of these, verifying the success of our approach. Reasons for our failure to recover the rest include (i) lack of the exogenous interface—known HMI—in our default template set due to small size of the interface such that it cannot meet our match criteria for structural alignment. Our template set includes exogenous interfaces having at least 15 residues and 1 hotspot on the non-human face of the interface, on which the oncoviral protein needs to be structurally aligned in order to interact with the complementary human-face. Some of the known exogenous interfaces have only a few residue-long non-human proteins in the crystal, such as EBNA1_EBV-UBP7_HUMAN interaction and cannot meet our match criteria for structural alignment, thus are discarded from the default template set. (ii) Some of the complexes in the crystal are multiprotein complexes, not binary interactions. A small pairwise interface may not be strong enough to stabilize the complex. For instance, BNRF1_EBV-DAXX_HUMAN is a multiprotein complex with histones H3.3 and H4, and the binary interaction interface without histones is not enough to give a favorable interaction energy and hence filtered from our results. (iii) Since chimeric proteins do not occur in nature, their interfaces are artificial as well and our template set do not have them. The chemokine receptor CXCR4 protein in K4_KSHV-CXCR4_HUMAN interface is a chimera of human and Enterobacteria phage T4. (iv) Another reason could be the input structure of the viral proteins. For instance, NS3_HCV can exist as isoforms of different sizes in distinct strains of the virus and which isoform was exploited as an input matters significantly. Our input structure for NS3 (PDB_ID: 3o8bA.pdb) is from HCV subtype 1b and is 628 residue-long. Whereas, the NS3 in NS3_HCV-MAVS_HUMAN exogenous interaction (PDB_ID: 3rc5AB.pdb) is from HCV subtype 1a and has 197 residues. The site where MAVS binds in one isoform is occupied by the rest of NS3 residues in the other isoform and therefore our method couldn't find the HMI due to steric clash. All in all, we can recover almost 100% of the structurally known HMIs if their interfaces are big enough to be included in our template set and if the input structure of the viral protein is of similar size and covers the same part of the viral protein in the template interface.

**Figure 3:**
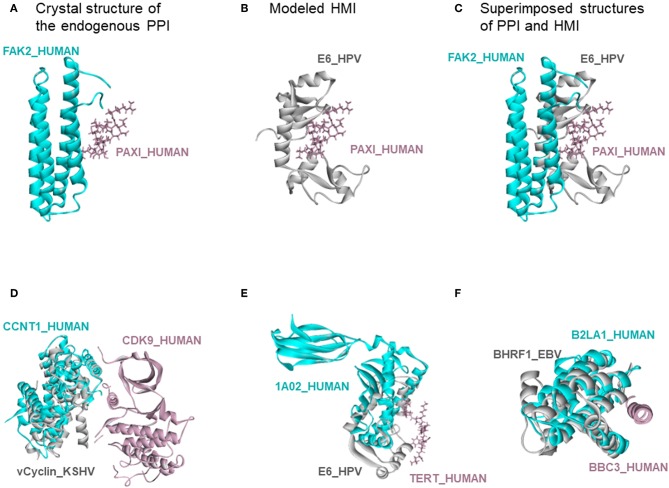
Recovery of known HMIs that do not have resolved complex structures. **(A)** Endogenous human protein interaction between FAK2 and PAXI. **(B)** Our E6_HPV-PAXI_HUMAN HMI model. **(C)** Superimposed structure of endogenous protein interaction and HMI shows that E6 mimics the interface on FAK2 to bind to PAXI. **(D)**, **(E)**, and **(F)** also show the superimposed views of endogenous human protein interaction and our HMI model. Cyan and pink proteins are human proteins; gray proteins are oncoviral proteins. Gray proteins interact with pink proteins by mimicking the interface on cyan proteins (only the interface is similar, despite different global structures). Therefore, they may block the pink-cyan protein interactions.

There are also 318 known host-oncovirus interactions in the databases, which do not have available complex structures (Table S5). It is not known whether these interactions are direct, or via bridging adaptors. Also, databases frequently compile data by text mining which may have errors. We have 9 common HMIs with these 318 interactions. Our method unearthed the complex structures of these 9 HMIs. Figure 3 shows some examples for these HMIs. Reasons why we could not find the rest of the HMIs in the databases may include (i) interactions may be indirect; (ii) partners in the host may not have resolved structures; (iii) even if they do have structures, they may not cover the full-length proteins; or (iv) they may not have interfaces in those structures (monomeric protein in the crystal). We complemented the HMI structural space, which has had very scarce data so far.

### Novel HMI Models and Their Cancer Hallmark Actions

When a virus enters the host, it needs to avoid clearance by the immune system, prevent host cell death, and ensure its latent persistence. In addition to recovering experimentally known interactions, our method also reveals many unknown ones which may relate to these actions. Below we provide examples in the context of cancer hallmarks, highlighting the importance of structures in unraveling the mechanistic basis in acquisition of these traits.

#### Evading the Host Immune Response

**Figure 4:**
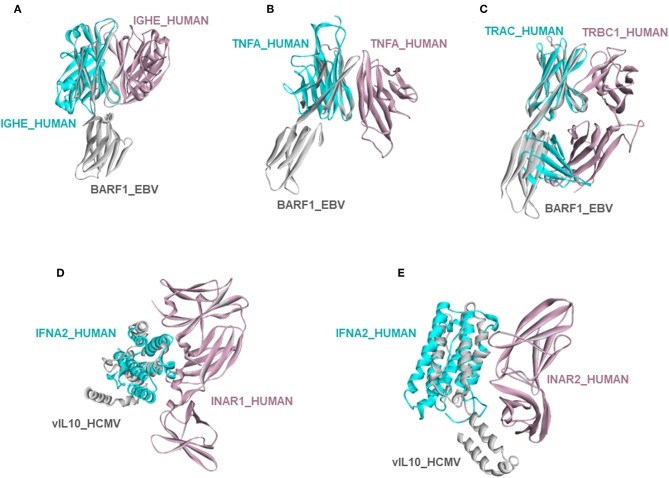
Examples of novel HMI models. **(A–C)** EBV secreted protein BARF1 hijacking the interfaces of host immune system regulators. **(D,E)** HCMV cytokine mimic vIL10 targets interferon receptors INAR1 and INAR2. All examples are shown as the superimposed views of endogenous human interactions and our HMI models.

Circumventing host immune recognition is the most important aim for pathogens. It is also vital for precancerous cells. We detected many immune-related proteins as targets of oncoviruses. For instance, secreted BARF1_EBV targets immunoglobulin constant heavy chains (IGHE), tumor necrosis factor (TNFA), and T-cell receptor beta 1 chain C region (TRBC1) (Figures 4A–C). Dimerization of immunoglobulin chains is necessary for antigen recognition. Our model suggests that BARF1_EBV interferes with dimerization. TNFA is a proinflammatory cytokine, produced mainly by immune cells. The active symmetric trimer TNFA binds its trimeric receptor. We found that BARF1_EBV ablates dimerization, thus TNFA trimerization. Moreover, BARF1_EBV also abrogates the heterodimerization of TCR α and β chains, impairing antigen recognition by T-cells. These results suggest that, via only BARF1 protein, EBV can intrude on host defense in different ways. EBV infection persists for decades and these potential HMIs may explain how the virus can evade the immune system. Without the complex structures and information of potentially affected human PPIs, it would be hard to understand the mechanistic basis of immune subversion.

Cytokine and chemokine signaling is an indispensable part of the host immunity and a frequent target of viral proteins. Viral chemokine and cytokine mimics, such as K4_KSHV, K6_KSHV, vIL6_KSHV, vIL10_HCMV, and vIL10_EBV can potentially heterodimerize with numerous host chemokines, chemokine and cytokine receptors, dampening or activating propagation of the signal through these key players. Despite not being cytokine or chemokine mimics, other viral proteins also confound cytokine and chemokine pathways: EBNA2_EBV can potentially bind to chemokine CCL16 and chemokine receptor CXCR4 and L1_HPV to cytokine IRF3 and cytokine receptor IL7RA.

Besides these, our models show that viral proteins are capable of multitasking/moonlighting. They may modulate alternative, non-canonical cytokine pathways in the host defense system. For instance, in addition to binding their canonical receptors, viral cytokines—vIL10_HCMV, vIL10_EBV, and vIL6_KSHV, which are known to bind to host IL10R and IL6R, respectively—also target other interleukin and interferon receptors, such as interferon alpha/beta receptor 1 (INAR1) and INAR2, to thwart binding of host interferons to this receptor (Figures 4D,E). Interferons are the main antiviral cytokines. Modulation of additional host cytokine receptors by viral cytokines may allow more efficient suppression of the host immunity.

We calculate the interaction energy [Rosetta interface score (I_sc) (31)] for both the HMI model and the template host PPI. Even though the energy function is empirical, the results may point which viral protein may outcompete the physiological cellular partner and bind to its target. We found that UL18_HCMV, a viral major histocompatibility complex (MHC) mimic, binds to β2-microglobulin (B2MG), by mimicking the interface on HLA class I histocompatibility antigen A-3 alpha chain (1A03) in the B2MG-1A03 complex (PDB_ID:2xpgAB). UL18_HCMV has much lower I_sc and thus higher affinity to B2MG than its endogenous competitor 1A03 (Table S1). 1A03 and B2MG are components of the MHC and interference of this complex by the viral protein may prevent the antigen presentation to T-cells. host immunity.

#### Sustaining Proliferative Signaling

Oncoviruses may promote survival and proliferation of host cells by modulating their cancer-related pathways. Comprehensive analysis of TCGA data (38) underscored 10 pathways that are mutated at higher frequencies in non-virally induced cancers and compiled the list of important genes in these pathways. These pathways are cell cycle, Hippo, Myc, Notch, Nrf2, PI3K/Akt, RTK-Ras, TGFβ, P53, and β-catenin/Wnt. We observed 82 common proteins within these highly mutated pathways that are oncoviral targets (Table S6). Examples of the proteins in these pathways are cyclins and cyclin-dependent kinases (CDKs) (cell cycle); MYC, MAX, MAD1 (Myc pathway); SKP1 and RBX1 (Notch pathway); MDM2, MDM4, and CHK2 (P53 pathway); PK3CA, MTOR, and PTEN (PI3K pathway); and JAK2 and PP1A (RTK-Ras pathway). CDKs and tyrosine protein kinase JAK2 act as cellular oncoproteins and MAX as a tumor suppressor. HMIs involving these proteins may increase the proliferative potential of the host cell. For example, cyclins are expressed only in certain phases of the cell cycle. CDK activation and cell cycle entry depends on the presence of cyclins. Viral proteins may substitute cyclins and overcome the requirement of expression of cellular cyclins to initiate cell division in the host cell. MAX is a transcription factor and can form homo- and hetero-dimers to initiate transcription. Depending on its binding partner, MYC or MAD1, the heterodimers can result in cell proliferation, differentiation, cell death, or quiescence (39). However, its homodimers have transcriptional repressive role because MAX lacks the transactivation domain (40). Homo- and hetero-dimers compete for the same site—E-box—on the DNA. We found that gL_EBV can bind to MAX by mimicking its interactions with MYC and MAD1.

There are other cell-cycle associated proteins among the targets of oncoproteins. CDK2-associated protein 1 (CDKA1) is a tumor suppressor and an inhibitor of CDK2. We found that E2_HPV, vIL10_ EBV, gB_HCMV, and vIL10_ HCMV proteins interfere with CDKA1 dimerization. Homodimer is the active form of CDKA1 since inhibition of dimer formation by the C105A mutation releases CDK2 inhibition (41). By preventing CDKA1 dimerization, i.e., inhibiting the inhibitor of CDK2, viral proteins may allow activation of CDK2 constitutively.

#### Promoting Cancer Through Activation of Human Oncogenes and Inhibition of Tumor Suppressors

Virus-targeted host proteins are enriched in oncogenes and tumor suppressors. This enrichment is statistically significant, with a *p*-value of 3.54e-23. 202 of the viral targets are listed as oncogenes and tumor suppressors in COSMIC Cancer Gene Census (release v85, 8th May 2018) (Table S7). As an example for oncogenes, deregulation of fibroblast growth factor (FGF) signaling and continuous activation of FGFRs, both ligand-dependent and ligand-independent (due to activating mutations, gene amplification, and gene fusion), promotes cancer development (42). FGFRs are targeted by 11 oncoviral proteins, mimicking FGF binding to these receptors which may increase the proliferative capacity of the host cells. The BRCA1-BARD1 complex serves as a good example for virus targeted tumor suppressors. This stable heterodimer complex has an E3 ubiquitin ligase activity, with important roles in genome stability, DNA repair, cell-cycle, and transcription (43). Both BRCA1 and BARD1 have low ubiquitin ligase activity on their own, but their heterodimer has much higher enzymatic activity (44). Several mutations seen in different cancers lie on the dimerization interface (45), such as C61G on BRCA1, abolishing the ubiquitin ligase activity (44). This enzymatic activity is critical in prevention of tumorigenesis. We found that the E2_HPV and p7_HCV proteins may interfere with the BARD1-BRCA1 heterodimer, potentially weakening its enzymatic action. This could result in uncontrolled proliferation. Without the HMI structures, which provide binding site information and the impacted endogenous host PPIs, the list of only the targets of the viruses would not explain how and why these virus-host interactions could contribute to the proliferative potential of the host cells.

#### Resisting Cell Death

In addition to modulating host immunity and cell cycle, oncoviruses also induce anti-apoptotic effects (46). Our models suggest that oncoviral proteins target numerous nodes in the apoptosis pathway. For example, p7_HCV, E7_HPV, and L1_HPV potentially bind to the death receptor, tumor necrosis factor receptor superfamily member 6 (TNR6, Fas) abolishing its interaction with Fas-associated death domain (FADD), which is required for the initiation of apoptotic signaling. In addition to impairing the recruitment of FADD to Fas receptor, oncoviral proteins also prevent dimerization of several caspases, which is necessary for their activation and triggering apoptosis. Pro-apoptotic proteins BAX, BAK, BIM (B2L11), BID, and BECN1 are also among the targets of oncoviruses. Anti-apoptotic MCL1 is a major resistance factor in chemotherapy (47, 48), and essential in breast cancer development (49). It inhibits pro-apoptotic proteins by directly interacting with them. We found that EBV apoptosis regulator protein BHRF1_EBV and other oncoviral proteins mimic the interactions of MCL1 with BID, BAX, and BIM, possibly inhibiting these apoptotic proteins, thus promoting survival of infected host cells. HUWE1 is an E3 ubiquitin ligase targeting MCL1 for degradation. Oncoviruses can also disrupt HUWEI-MCL1 interaction, preventing MCL1 degradation.

TRAIL (TNF10) signaling is one of the three major pathways inducing apoptosis. Binding of symmetric TRAIL trimers to the trimeric receptors recruits FADD and Capase8 to form death inducing signaling complex (DISC) and initiate apoptosis (50). gH_EBV binds to TRAIL, abolishing its interaction with its receptor TR10B and hence initiation of apoptosis.

#### Invasion and Metastasis

Oncoviruses may act not only during tumor initiation but also the progression and metastasis. Expression and signaling of cell-cell and cell-ECM (extracellular matrix) adhesion molecules are altered in aggressive tumors (51). Table S8 shows the KEGG pathways, enriched with oncovirus-targeted host proteins. Several oncoviruses attack cell adhesion, focal adhesion and adherence junction pathways at different nodes, including integrins. Integrins function as heterodimeric surface receptors that recognize ECM proteins and mediate cell-ECM adhesion, migration, and proliferation (52). Invasion from primary tumor site requires integrins, as their genetic depletion and pharmacological targeting reduces metastasis (53, 54). UNG_EBV and vIRF2_KSHV can bind to integrin alpha-IIb (ITA2B), hijacking ITA2B-ITB3 interaction. Integrin heterodimer complex with these viral proteins may be functional and enable metastasis.

Rho GTPases are critical orchestrators of cytoskeletal dynamics, cell motility, and metastasis (55). Rho proteins get activated by Rho guanine nucleotide exchange factors (RhoGEFs) and inactivated by Rho GTPase-activating proteins (RhoGAPs). The roles of RHOA in both initiation (56) and prevention of metastasis have been controversial (57, 58). Oncoviruses target several RhoGEFs, such as ARHGB, ARHGC, and ARHGP—mimicking their interactions with RHOA, as well as RHOA itself.

#### Genetic Instability

Abnormal telomere homeostasis has pivotal roles in genetic instability, which allows accumulation of mutations and ultimately malignant transformation (59). Telomeres are protected by “shelterin” protein complexes, disruption of which is highly toxic for cancer cells (60). Telomeric repeat-binding factor 1 (TERF1) and TERF2 are key factors in the shelterin complex and they need to homodimerize to associate with telomeric DNA (61). TERF2 depletion leads to chromosome end fusions, resulting in genome instability (62). Its overexpression has been reported in prostate (63), liver (64), and lung cancers (65). Its down-regulation is also seen in breast (66) and gastric cancers (67). Experimental induction of TERF2 overexpression has been shown to cause telomere shortening, independent of telomerase activity and subsequent chromosome fusions (62). Our results show that US2_HCMV, NS2_HCV, and E2_HPV may interact with TERF2, ablate TERF2 homodimerization, and prevent shelterin formation at the telomeres, which may in turn result in telomere erosion and genome instability.

#### Tumor Promoting Inflammation

Despite its critical roles in host defense and healing wounds, inflammation can also contribute to all steps of carcinogenesis (68, 69). Chronic inflammation is one of the main risk factors in virus-driven cancers (46). NF-κB is the major pro-inflammatory pathway. It is inactive when IκB, an inhibitor of NF-κB, sequesters and retains it in the cytoplasm. Upon phosphorylation by IKK complex, IκB is degraded and NF-κB translocates to nucleus and initiates transcription of inflammatory cytokines. It has been known that vFLIP_KSHV binds and activates IKKγ (NEMO), an indispensable part of the IKK complex, and leads to constitutive activation of the canonical NF-κB pathway (70). We observed that other oncoviral proteins, GP110_EBV, UNG_EBV, HBcAg_HBV, NS2_HCV, p7_HCV, and L1_HPV can associate with NEMO, mimicking its interaction with the β-subunit of the IKK complex (IKKB). Like vFLIP_KSHV, these viral proteins may also activate the IKK complex and render constitutive activation of NF-κB and chronic inflammation.

### Common Targets of Oncoviruses

**Figure 5:**
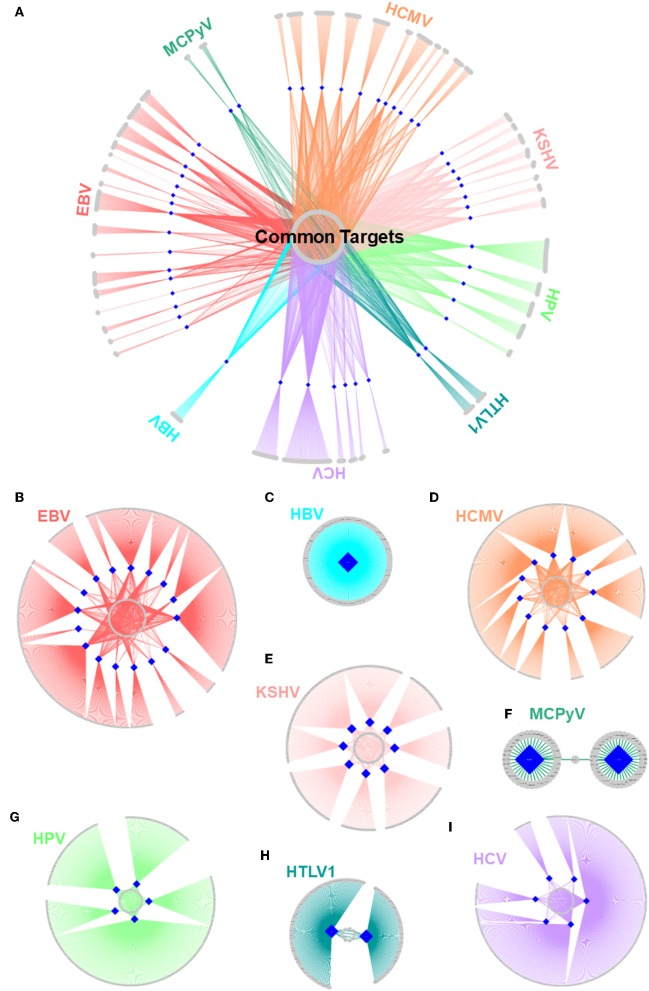
Structural HMI networks. **(A)** Structural HMI network for all known oncoviruses. Most of the targets of viral proteins are unique, but there are also some common ones (central part) across different viruses. Human proteins are shown as gray circular nodes and oncoviral proteins are blue diamond-shaped nodes. **(B–I)** Structural HMI networks for individual oncoviruses. Viruses can target the same host protein with more than 1 of its proteins (middle parts of the circles: some host proteins are common targets to more than 1 protein).

With all potential HMIs in hand, it is possible to evaluate the common trends among oncoviruses. Although most host proteins are unique targets for certain viral proteins, there are also common ones, suggesting convergent evolution toward similar strategies to attack the host (Figure 5A). Table S9 provides the list of frequently targeted host proteins. Among these, there are Polyubiquitin-c (UBC), UBB, and E3 ubiquitin ligase MDM2. Ubiquitin pathway regulates diverse biological processes, including cell growth, cell death, and immunity. Both viruses (71) and cancer cells (72) exploit the ubiquitin system to maximize their survival, by either stabilizing the negative regulators of apoptosis and immunity or deregulating the main actors of these pathways. MDM2 is a negative regulator of P53 and overexpressed in many cancers. It functions as a homodimer or heterodimers with MDM4 and MDMX. Mutants incapable of heterodimerization fail to restrict P53 function (73). Our results show that MDM2 can be targeted by 17 proteins, from 6 oncoviruses. Heterodimerization with viral proteins may lead to enzymatic activation of MDM2 and proteasomal degradation of P53.

### An Oncovirus May Target a Host Protein or a Host Pathway With Several Proteins

There are also cases in which an oncovirus targets a host protein with several proteins (Figures 5B–I). This is not surprising because high-throughput yeast-2-hybrid experiments for Vaccinia virus also identified host proteins with more than one viral binding partner (74). Humans also have a back-up mechanism (i.e., compensatory microbial sensing), with several pattern recognition receptors recognizing the same microbial antigen (75). Viruses can target the same (overlapping) or distinct (non-overlapping) interfaces on the host protein. For instance, 5 EBV proteins potentially interact with retinoic acid receptor RXR-alpha (RXRA)—some through the same interface and others via a distinct interface. Since proteins may not be expressed at all phases of the viral cell cycle, it is reasonable that a virus possesses more than one protein to target the same host protein to support persistent inhibition (or activation). Spatiotemporal distribution, host cell/tissue type, and subcellular location may also be involved.

Table S8 shows that potential oncoviral host targets are enriched in KEGG pathways for corresponding oncoviruses. For instance, “Epstein-Barr virus infection” KEGG pathway is the most enriched pathway (with lowest *p*-value) in EBV targeted host proteins and “Hepatitis B” pathway is the fourth significant pathway in HBV-targeted proteins. This further indicates the success of our method to correctly identify the host proteins that are involved in viral infections.

Virus-targeted host proteins are also enriched in other KEGG pathways, suggesting their pleiotropic effects in the host. Since tumorigenesis is a multistep process, it may require inhibition/activation of diverse set of pathways. Along these lines, our observations also suggest that viral proteins can act redundantly, attacking a pathway at more than one node. For instance, EBV targets 23 different nodes in the Toll-like receptor signaling pathway, which is key in innate immunity. Furthermore, frequently targeted pathways (Table S10) suggest that EBV, HBV, HCV and HCMV may have convergently evolved to attack the same host pathways even though these viruses differ. Sixty-four KEGG pathways, including apoptosis, TLR, MAPK, and PI3K-Akt, are common to these four viruses. A therapeutic approach against a particular oncovirus may be expanded to others if they have common targets and share similar infection mechanisms.

### Therapeutic Actionability of the Oncovirus-Targeted Host Proteins

It is also possible to assess the therapeutic actionability of oncovirus-targeted host proteins. We observed 53 FDA-approved anticancer drugs (76) against 57 of the virus-targeted host proteins (Table S11). For instance, BRAF is targeted by 2 viral proteins (NS2_HCV and E7_HPV) and it has 4 chemotherapy drugs that are used in different non-virally induced cancers. Another example would be estrogen receptors (ESR1 and ESR2), against which 5 drugs were developed to cure breast and prostate cancers. Although these anticancer agents have not been used in virus-driven cancers, they could be repurposed.

### Structural Superorganism Network

**Figure 6:**
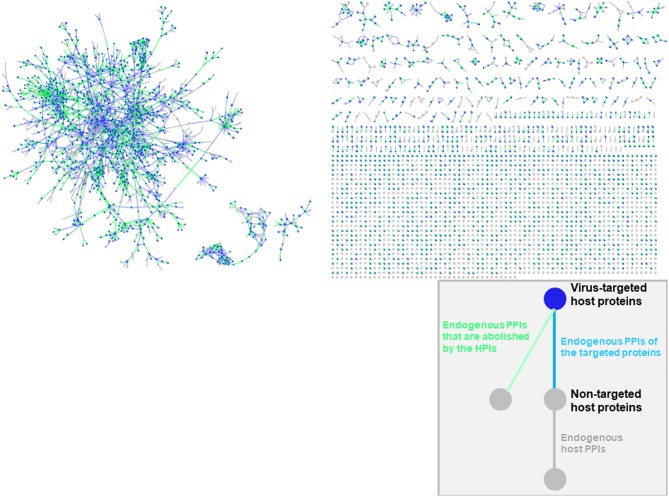
Structural inter-species interaction network for all oncoviruses. Oncoviral proteins target the hub proteins of the human PPI network. For simplicity, we haven't showed the oncoviral proteins and their interactions with host proteins here, but only the host proteins with green, blue or gray highlighted edges. All pairwise interactions have structures as complexes in this network. Endogenous human PPIs are obtained from available crystal structures (template interface set).

Oncoviral tumorigenicity cannot be explained by individual oncovirus-host interactions, as they have complex and dynamic interaction profiles. Viral proteins cooperate to rewire the host pathways, endowing the host cell with multiple hallmark capabilities. The availability of the structures of HMI complexes allow constructing the rewired host-pathogen superorganism protein interaction network in structural detail. To date, there is no structural inter-species network that incorporates HMIs for all oncoviruses with endogenous host PPIs. The topological features of such networks can elucidate regulatory nodes or modules that the viruses target. As such, previously built superorganism networks revealed that bacteria and viruses generally target hub proteins (12, 77). We constructed a comprehensive structural superorganism network for all oncoviruses, which comprises all structurally known endogenous human PPIs and our HMI models (Figure 6). All pairwise interactions here have structures as complexes. There are 6,456 distinct endogenous human PPIs (our template set) and 6,034 distinct exogenous interactions (our HMIs) in the network. Oncoviral proteins target the highly-connected part of the network, suggesting modulation of multiple host pathways by the oncoviruses. Analysis of the topological features of the network showed that hub proteins, such as B2MG, UBC, UBB, HLA class I histocompatibility antigen A-2 alpha chain (1A02), Calmodulin-2 (CALM2), and T cell receptor beta constant 1 (TRBC1) are targeted by viral proteins. According to TCGA, these high connectivity nodes are also mutated in non-virally induced cancers. Viruses with only few proteins can perturb many cellular functions, as hub proteins mediate the crosstalk across several pathways.

## Discussion

Genome-wide experimental characterization of the interactions of microbe proteins with those of human, especially on the structural level, is challenging (78, 79). High throughput experimental techniques to detect HMIs include yeast-2-hybrid, protein arrays, and mass-spectrometry coupled with affinity purification or chemical crosslinking (11), but they cannot resolve structures. Also, each technique comes with its drawbacks, such as yeast-2-hybrid having a high rate of false negatives since it only detects interactions that take place in the yeast nucleus. Completion of the HMI space requires robust computational techniques (78, 80, 81). Computational screening of big data can guide experiments by providing possible leads and bypass testing millions of possible pairwise combinations of host and microbial proteins. Predictions of the computational methods should be validated by experiments. Homology-based methods are useful only if the sequence similarity is very high. However, many microbial proteins do not have homologs in human. Also, global structure similarity-based methods can identify fewer HMIs compared to interface-based methods, since interface mimicry is much more frequent than global structure similarity. Interface-based approaches can be applied on a large-scale, holding promise to enrich the HMI space.

We developed a powerful interface-based HMI prediction method and a user-friendly HMI-PRED webserver, which can be applied to pathogens or commensals (25). It is the first and currently only interface-based HMI modeling approach. The outputs are the list of HMIs and their structures as bound complexes; list of mimicked/disrupted endogenous human PPIs; and list of exogenous PPIs with other microbes that are mimicked by the microbe of interest, suggesting convergent evolution of distinct microbes having common targets. We demonstrated the utility of our approach by applying it to the oncobacterium *Helicobacter pylori* (24).

Studying HMIs one-at-a-time may not uncover accurately the tumorigenic mechanisms of oncoviruses. Combinatorial effects of distinct HMIs as well as simultaneously active/suppressed host pathways will determine the type and magnitude of the cellular response. Integrated superorganism networks that consider the microbe and the host interactions as a whole, are useful in identifying the key regulatory nodes or modules (13). Topological features of such networks can delineate the roles of pathogen-targeted host proteins in the network, with hub and bottleneck nodes appearing to be the main targets (12, 77, 82). A superorganism network that combines interactions of the microbes with the host proteins, as well as the endogenous host interactions, along with their structural details, are more useful than the schematic “node-and-edge” network diagrams. Structural networks can reveal how targeting one endogenous host interface will affect the whole system, as it can disturb all interactions which exploit similar interfaces (83). We built an integrated structural network for oncoviruses and their human host, where all pairwise interactions have structures. We observed that some hub proteins such as UBC, UBB, B2MG, A102, CALM2, and TRBC1 are among the potential targets of oncoviruses.

The availability of structures can also facilitate drug discovery. For instance, poxviruses utilize host Abl and Src kinases in their life cycle and Gleevec, an anticancer drug against Abl family kinases, mitigates poxvirus infection mortality (84). To date, attempts to decrease pathogen-driven cancer incidents mainly aim to extinguish the viral infection before the onset of cancer. There are vaccines against HPV and HBV, which have been very effective in reducing the infection rates, hence incidents of cervical and hepatocellular carcinoma (6). However, these vaccines are not therapeutics. They do not provide benefits to treat established cancers. Therapies for virus-induced cancers remain limited. Exploring the underlying molecular mechanisms and identifying novel HMIs can innovate therapeutic and prophylactic intervention. Identifying druggable viral targets is an attractive research area in *de novo* drug design. There is also opportunity for drug repurposing, if the oncovirus-targeted host proteins already have FDA approved drugs for other cancer types. There are 53 FDA-approved anti-cancer drugs against 57 of the oncovirus-targeted host proteins that we found. Complete list of HMIs also favors identification of synergistic drug combinations.

It is also important to note the caveats of interface-based approaches, such as low coverage of protein interactions (85), particularly transient interactions, and underrepresentation of disordered proteins in the PDB (86, 87), which limits the diversity of the template set. Success of interface-based methods depends on the quality and completeness of the templates (88). Still, the available interface structures are suggested to be diverse enough to cover most endogenous protein interactions (89, 90) indicating that interface-based methods can capture most of the interactions. Another hurdle is the lack of 3D structures for most pathogenic proteins, which is the only input for our method. This can be overcome with recent advancements in ab initio modeling of unknown structures (91) and providing homology models as inputs to our method.

Since thousands of microbial species inhabit the human host, making it a “metaorganism,” interactions of pathogens with the inhabitant microbiota may also affect the overall response. Moreover, proteins often form multi-protein complexes, rather than binary complexes. Also, protein interactions are not the only interaction type: viruses can interact with the host through nucleic acids, such as miRNAs, as well. Therefore, for a broader view of the viral impacts on human hosts, interactions with microbiota, interactions through other molecules and multi-protein complexes should also be considered.

In conclusion, it has long been known that viruses can trigger tumorigenesis in humans, but to date, the exact molecular mechanisms have still been unclear. Here, we have elucidated possible molecular events that may occur in oncovirus infected host cells. Our results reveal the structural basis for how host cells may attain cancer hallmark traits through their interactions with oncoviral proteins, and these mimic those presented by the non-virally induced cancer. This has been expected in the community; and here we verify this expectation and show how.

These results testify to the advantages of computational approaches and argue that despite their inherent limitations, large-scale characterization of these interactions benefit from large-scale computational approaches. The next step should involve experimental testing and structural determination of the new predictions to verify the interaction and optimize the therapeutics. Further computational software developments and data are also sorely needed.

## Data Availability Statement

The 3D structures of the oncoviral proteins analyzed in this study are available in Protein Data Bank (PDB). The complex (bound) structures of all HMIs that we modeled here is available in HMI-PRED webserver under the “Predictions” tab, which is a repository to store all previous results. All datasets generated for this study are included in the article/Supplementary Material.

## Author Contributions

EG-M, C-JT, and RN conceived and designed the study. EG-M carried out the predictions of the HMIs between oncoviruses and human, analyzed the data, and wrote the manuscript. All authors edited the manuscript.

### Conflict of Interest

The authors declare that the research was conducted in the absence of any commercial or financial relationships that could be construed as a potential conflict of interest.

## References

[1] Akram N Imran M Noreen M Ahmed F Atif M Fatima Z. Oncogenic role of tumor viruses in humans. Viral Immunol. (2017) 30:20–7. 10.1089/vim.2016.0109 27830995

[2] Dalton-Griffin L Kellam P. Infectious causes of cancer and their detection. J Biol. (2009) 8:67. 10.1186/jbiol168 19678917 PMC2736673

[3] Mui UN Haley CT Tyring SK. Viral oncology: molecular biology and pathogenesis. J Clin Med. (2017) 6:E111. 10.3390/jcm6120111 29186062 PMC5742800

[4] IARC Working Group on the Evaluation of Carcinogenic Risks to Humans Volume 100 B. A review of human carcinogens. IARC Monogr Eval Carcinog Risks Hum. (2012) 100(Pt B):1–441. PMC4781184 23189750

[5] McLaughlin-Drubin ME Munger K. Viruses associated with human cancer. Biochim Biophys Acta. (2008) 1782:127–50. 10.1016/j.bbadis.2007.12.005 18201576 PMC2267909

[6] Vandeven N Nghiem P. Pathogen-driven cancers and emerging immune therapeutic strategies. Cancer Immunol Res. (2014) 2:9–14. 10.1158/2326-6066.CIR-13-0179 24778160 PMC4135058

[7] Melnick M Sedghizadeh PP Allen CM Jaskoll T. Human cytomegalovirus and mucoepidermoid carcinoma of salivary glands: cell-specific localization of active viral and oncogenic signaling proteins is confirmatory of a causal relationship. Exp Mol Pathol. (2012) 92:118–25. 10.1016/j.yexmp.2011.10.011 22101257

[8] Moore PS Chang Y. Why do viruses cause cancer? Highlights of the first century of human tumour virology. Nat Rev Cancer. (2010) 10:878–89. 10.1038/nrc2961 21102637 PMC3718018

[9] Wang Q Xu Y Zhou W Zhong L Wen Z Yu H. The viral oncoprotein HBx of Hepatitis B virus promotes the growth of hepatocellular carcinoma through cooperating with the cellular oncoprotein RMP. Int J Biol Sci. (2014) 10:1181–92. 10.7150/ijbs.10275 25516716 PMC4261202

[10] White MK Pagano JS Khalili K. Viruses and human cancers: a long road of discovery of molecular paradigms. Clin Microbiol Rev. (2014) 27:463–81. 10.1128/CMR.00124-13 24982317 PMC4135891

[11] Nicod C Banaei-Esfahani A Collins BC. Elucidation of host-pathogen protein-protein interactions to uncover mechanisms of host cell rewiring. Curr Opin Microbiol. (2017) 39:7–15. 10.1016/j.mib.2017.07.005 28806587 PMC5732060

[12] Franzosa EA Xia Y. Structural principles within the human-virus protein-protein interaction network. Proc Natl Acad Sci USA. (2011) 108:10538–43. 10.1073/pnas.1101440108 21680884 PMC3127880

[13] Guven-Maiorov E Tsai CJ Nussinov R. Structural host-microbiota interaction networks. PLoS Comput Biol. (2017) 13:e1005579. 10.1371/journal.pcbi.1005579 29023448 PMC5638203

[14] Guven-Maiorov E Tsai CJ Nussinov R. Pathogen mimicry of host protein-protein interfaces modulates immunity. Semin Cell Dev Biol. (2016) 58:136–45. 10.1016/j.semcdb.2016.06.004 27287306

[15] Keskin O Nussinov R. Similar binding sites and different partners: implications to shared proteins in cellular pathways. Structure. (2007) 15:341–54. 10.1016/j.str.2007.01.007 17355869

[16] Keskin O Nussinov R. Favorable scaffolds: proteins with different sequence, structure and function may associate in similar ways. Protein Eng Des Sel. (2005) 18:11–24. 10.1093/protein/gzh095 15790576

[17] Tsai CJ Lin SL Wolfson HJ Nussinov R. A dataset of protein-protein interfaces generated with a sequence-order-independent comparison technique. J Mol Biol. (1996) 260:604–20. 10.1006/jmbi.1996.0424 8759323

[18] Tsai CJ Lin SL Wolfson HJ Nussinov R. Protein-protein interfaces: architectures and interactions in protein-protein interfaces and in protein cores. Their similarities and differences. Crit Rev Biochem Mol Biol. (1996) 31:127–52. 10.3109/10409239609106582 8740525

[19] Guven-Maiorov E Keskin O Gursoy A VanWaes C Chen Z Tsai CJ. TRAF3 Signaling: competitive binding and evolvability of adaptive viral molecular mimicry. Biochim Biophys Acta. (2016) 1860(11 Pt B):2646–55. 10.1016/j.bbagen.2016.05.021 27208423 PMC7117012

[20] Franzosa EA Garamszegi S Xia Y. Toward a three-dimensional view of protein networks between species. Front Microbiol. (2012) 3:428. 10.3389/fmicb.2012.00428 23267356 PMC3528071

[21] Chow D He X Snow AL Rose-John S Garcia KC. Structure of an extracellular gp130 cytokine receptor signaling complex. Science. (2001) 291:2150–5. 10.1126/science.1058308 11251120

[22] Qin L Kufareva I Holden LG Wang C Zheng Y Zhao C. Structural biology. Crystal structure of the chemokine receptor CXCR4 in complex with a viral chemokine. Science. (2015) 347:1117–22. 10.1126/science.1261064 25612609 PMC4362693

[23] Yoon SI Jones BC Logsdon NJ Walter MR. Same structure, different function crystal structure of the Epstein-Barr virus IL-10 bound to the soluble IL-10R1 chain. Structure. (2005) 13:551–64. 10.2210/pdb1y6m/pdb 15837194

[24] Guven-Maiorov E Tsai CJ Ma B Nussinov R. Prediction of host-pathogen interactions for helicobacter pylori by interface mimicry and implications to gastric cancer. J Mol Biol. (2017) 429:3925–41. 10.1016/j.jmb.2017.10.023 29106933 PMC7906438

[25] Guven-Maiorov E Tsai CJ Ma B Nussinov R. Interface-based structural prediction of novel host-pathogen interactions. Methods Mol Biol. (2019) 1851:317–35. 10.1007/978-1-4939-8736-8_18 30298406 PMC8192064

[26] Cukuroglu E Gursoy A Nussinov R Keskin O. Non-redundant unique interface structures as templates for modeling protein interactions. PLoS ONE. (2014) 9:e86738. 10.1371/journal.pone.0086738 24475173 PMC3903793

[27] Tuncbag N Gursoy A Nussinov R Keskin O. Predicting protein-protein interactions on a proteome scale by matching evolutionary and structural similarities at interfaces using PRISM. Nat Protoc. (2011) 6:1341–54. 10.1038/nprot.2011.367 21886100 PMC7384353

[28] Keskin O Nussinov R Gursoy A. PRISM: protein-protein interaction prediction by structural matching. Methods Mol Biol. (2008) 484:505–21. 10.1007/978-1-59745-398-1_30 18592198 PMC2685641

[29] Baspinar A Cukuroglu E Nussinov R Keskin O Gursoy A. PRISM: a web server and repository for prediction of protein-protein interactions and modeling their 3D complexes. Nucleic Acids Res. (2014) 42:W285–9. 10.1093/nar/gku397 24829450 PMC4086120

[30] Ogmen U Keskin O Aytuna AS Nussinov R Gursoy A. PRISM: protein interactions by structural matching. Nucleic Acids Res. (2005) 33:W331–6. 10.1093/nar/gki585 15991339 PMC1160261

[31] Wang C Bradley P Baker D. Protein-protein docking with backbone flexibility. J Mol Biol. (2007) 373:503–19. 10.1016/j.jmb.2007.07.050 17825317

[32] Duarte JM Srebniak A Scharer MA Capitani G. Protein interface classification by evolutionary analysis. BMC Bioinformatics. (2012) 13:334. 10.1186/1471-2105-13-334 23259833 PMC3556496

[33] Uhlen M Bjorling E Agaton C Szigyarto CA Amini B Andersen E. A human protein atlas for normal and cancer tissues based on antibody proteomics. Mol Cell Proteomics. (2005) 4:1920–32. 10.1074/mcp.R500009-MCP200 16127175

[34] Shannon P Markiel A Ozier O Baliga NS Wang JT Ramage D. Cytoscape: a software environment for integrated models of biomolecular interaction networks. Genome Res. (2003) 13:2498–504. 10.1101/gr.1239303 14597658 PMC403769

[35] Yang H Ke Y Wang J Tan Y Myeni SK Li D. Insight into bacterial virulence mechanisms against host immune response via the Yersinia pestis-human protein-protein interaction network. Infect Immun. (2011) 79:4413–24. 10.1128/IAI.05622-11 21911467 PMC3257920

[36] Huang da W Sherman BT Lempicki RA. Bioinformatics enrichment tools: paths toward the comprehensive functional analysis of large gene lists. Nucleic Acids Res. (2009) 37:1–13. 10.1093/nar/gkn923 19033363 PMC2615629

[37] Huang da W Sherman BT Lempicki RA. Systematic and integrative analysis of large gene lists using DAVID bioinformatics resources. Nat Protoc. (2009) 4:44–57. 10.1038/nprot.2008.211 19131956

[38] Sanchez-Vega F Mina M Armenia J Chatila WK Luna A La KC. Oncogenic signaling pathways in the cancer genome Atlas. Cell. (2018) 173:321–37.e10. 10.1016/j.cell.2018.03.035 29625050 PMC6070353

[39] Nair SK Burley SK. X-ray structures of Myc-Max and Mad-Max recognizing DNA. Molecular bases of regulation by proto-oncogenic transcription factors. Cell. (2003) 112:193–205. 10.1016/S0092-8674(02)01284-9 12553908

[40] Farina A Faiola F Martinez E. Reconstitution of an E box-binding Myc:max complex with recombinant full-length proteins expressed in *Escherichia coli*. Protein Expr Purif . (2004) 34:215–22. 10.1016/j.pep.2003.11.021 15003254 PMC4004042

[41] Ertekin A Aramini JM Rossi P Leonard PG Janjua H Xiao R. Human cyclin-dependent kinase 2-associated protein 1 (CDK2AP1) is dimeric in its disulfide-reduced state, with natively disordered N-terminal region. J Biol Chem. (2012) 287:16541–9. 10.1074/jbc.M112.343863 22427660 PMC3351331

[42] Saichaemchan S Ariyawutyakorn W Varella-Garcia M. Fibroblast growth factor receptors: from the oncogenic pathway to targeted therapy. Curr Mol Med. (2016) 16:40–62. 10.2174/1566524016666151222144231 26695695

[43] Baer R Ludwig T. The BRCA1/BARD1 heterodimer, a tumor suppressor complex with ubiquitin E3 ligase activity. Curr Opin Genet Dev. (2002) 12:86–91. 10.1016/S0959-437X(01)00269-6 11790560

[44] Hashizume R Fukuda M Maeda I Nishikawa H Oyake D Yabuki Y. The RING heterodimer BRCA1-BARD1 is a ubiquitin ligase inactivated by a breast cancer-derived mutation. J Biol Chem. (2001) 276:14537–40. 10.1074/jbc.C000881200 11278247

[45] Brzovic PS Rajagopal P Hoyt DW King MC Klevit RE. Structure of a BRCA1-BARD1 heterodimeric RING-RING complex. Nat Struct Biol. (2001) 8:833–7. 10.1038/nsb1001-833 11573085

[46] Mesri EA Feitelson MA Munger K. Human viral oncogenesis: a cancer hallmarks analysis. Cell Host Microbe. (2014) 15:266–82. 10.1016/j.chom.2014.02.011 24629334 PMC3992243

[47] Lee KM Giltnane JM Balko JM Schwarz LJ Guerrero-Zotano AL Hutchinson KE. MYC and MCL1 cooperatively promote chemotherapy-resistant breast cancer stem cells via regulation of mitochondrial oxidative phosphorylation. Cell Metab. (2017) 26:633–47.e7. 10.1158/1538-7445.AM2016-3328 28978427 PMC5650077

[48] Michels J Obrist F Vitale I Lissa D Garcia P Behnam-Motlagh P. MCL-1 dependency of cisplatin-resistant cancer cells. Biochem Pharmacol. (2014) 92:55–61. 10.1016/j.bcp.2014.07.029 25107702

[49] Campbell KJ Dhayade S Ferrari N Sims AH Johnson E Mason SM. MCL-1 is a prognostic indicator and drug target in breast cancer. Cell Death Dis. (2018) 9:19. 10.1038/s41419-017-0035-2 29339815 PMC5833338

[50] Liu H Su D Zhang J Ge S Li Y Wang F. Improvement of pharmacokinetic profile of TRAIL via trimer-tag enhances its antitumor activity *in vivo*. Sci Rep. (2017) 7:8953. 10.1038/s41598-017-09518-1 28827692 PMC5566391

[51] Farahani E Patra HK Jangamreddy JR Rashedi I Kawalec M Rao Pariti RK. Cell adhesion molecules and their relation to (cancer) cell stemness. Carcinogenesis. (2014) 35:747–59. 10.1093/carcin/bgu045 24531939

[52] Seguin L Desgrosellier JS Weis SM Cheresh DA. Integrins and cancer: regulators of cancer stemness, metastasis, and drug resistance. Trends Cell Biol. (2015) 25:234–40. 10.1016/j.tcb.2014.12.006 25572304 PMC4380531

[53] Li Y Drabsch Y Pujuguet P Ren J van Laar T Zhang L. Genetic depletion and pharmacological targeting of alphav integrin in breast cancer cells impairs metastasis in zebrafish and mouse xenograft models. Breast Cancer Res. (2015) 17:28. 10.1186/s13058-015-0537-8 25849225 PMC4381510

[54] Ganguly KK Pal S Moulik S Chatterjee A. Integrins and metastasis. Cell Adh Migr. (2013) 7:251–61. 10.4161/cam.23840 23563505 PMC3711990

[55] Jansen S Gosens R Wieland T Schmidt M. Paving the Rho in cancer metastasis: Rho GTPases and beyond. Pharmacol Ther. (2018) 183:1–21. 10.1016/j.pharmthera.2017.09.002 28911825

[56] Yang X Zheng F Zhang S Lu J. Loss of RhoA expression prevents proliferation and metastasis of SPCA1 lung cancer cells *in vitro*. Biomed Pharmacother. (2015) 69:361–6. 10.1016/j.biopha.2014.12.004 25661383

[57] Alkasalias T Alexeyenko A Hennig K Danielsson F Lebbink RJ Fielden M. RhoA knockout fibroblasts lose tumor-inhibitory capacity *in vitro* and promote tumor growth *in vivo*. Proc Natl Acad Sci USA. (2017) 114:E1413–21. 10.1073/pnas.1621161114 28174275 PMC5338371

[58] Rodrigues P Macaya I Bazzocco S Mazzolini R Andretta E Dopeso H. RHOA inactivation enhances Wnt signalling and promotes colorectal cancer. Nat Commun. (2014) 5:5458. 10.1038/ncomms6458 25413277 PMC4255233

[59] O'Sullivan RJ Karlseder J. Telomeres: protecting chromosomes against genome instability. Nat Rev Mol Cell Biol. (2010) 11:171–81. 10.1038/nrm2848 20125188 PMC2842081

[60] Bilsland AE Cairney CJ Keith WN. Targeting the telomere and shelterin complex for cancer therapy: current views and future perspectives. J Cell Mol Med. (2011) 15:179–86. 10.1111/j.1582-4934.2010.01253.x 21199331 PMC3822786

[61] Chen Y Yang Y van Overbeek M Donigian JR Baciu P de Lange T. A shared docking motif in TRF1 and TRF2 used for differential recruitment of telomeric proteins. Science. (2008) 319:1092–6. 10.1126/science.1151804 18202258

[62] Nera B Huang HS Lai T Xu L. Elevated levels of TRF2 induce telomeric ultrafine anaphase bridges and rapid telomere deletions. Nat Commun. (2015) 6:10132. 10.1038/ncomms10132 26640040 PMC4686832

[63] Chen W Wang Y Li F Lin W Liang Y Ma Z. Expression of telomere repeat binding factor 1 and TRF2 in prostate cancer and correlation with clinical parameters. BioMed Res Int. (2017) 2017:9764752. 10.1155/2017/9764752 28808664 PMC5541806

[64] Oh BK Kim YJ Park C Park YN. Up-regulation of telomere-binding proteins, TRF1, TRF2, and TIN2 is related to telomere shortening during human multistep hepatocarcinogenesis. Am J Pathol. (2005) 166:73–80. 10.1016/S0002-9440(10)62233-X 15632001 PMC1602303

[65] Nakanishi K Kawai T Kumaki F Hiroi S Mukai M Ikeda E. Expression of mRNAs for telomeric repeat binding factor (TRF)-1 and TRF2 in atypical adenomatous hyperplasia and adenocarcinoma of the lung. Clin Cancer Res. (2003) 9:1105–11. 12631614

[66] Saito K Yagihashi A Nasu S Izawa Y Nakamura M Kobayashi D. Gene expression for suppressors of telomerase activity (telomeric-repeat binding factors) in breast cancer. Jpn J Cancer Res. (2002) 93:253–8. 10.1111/j.1349-7006.2002.tb02166.x 11927006 PMC5926971

[67] Yamada M Tsuji N Nakamura M Moriai R Kobayashi D Yagihashi A. Down-regulation of TRF1, TRF2 and TIN2 genes is important to maintain telomeric DNA for gastric cancers. Anticancer Res. (2002) 22:3303–7. 12530079

[68] Guven Maiorov E Keskin O Gursoy A Nussinov R. The structural network of inflammation and cancer: merits and challenges. Semin Cancer Biol. (2013) 23:243–51. 10.1016/j.semcancer.2013.05.003 23712403

[69] Trinchieri G. Cancer and inflammation: an old intuition with rapidly evolving new concepts. Annu Rev Immunol. (2012) 30:677–706. 10.1146/annurev-immunol-020711-075008 22224761

[70] Bagneris C Ageichik AV Cronin N Wallace B Collins M Boshoff C. Crystal structure of a vFlip-IKKgamma complex: insights into viral activation of the IKK signalosome. Mol Cell. (2008) 30:620–31. 10.1016/j.molcel.2008.04.029 18538660

[71] Viswanathan K Fruh K DeFilippis V. Viral hijacking of the host ubiquitin system to evade interferon responses. Curr Opin Microbiol. (2010) 13:517–23. 10.1016/j.mib.2010.05.012 20699190 PMC2939720

[72] Senft D Qi J Ronai ZA. Ubiquitin ligases in oncogenic transformation and cancer therapy. Nat Rev Cancer. (2018) 18:69–88. 10.1038/nrc.2017.105 29242641 PMC6054770

[73] Pant V Xiong S Iwakuma T Quintas-Cardama A Lozano G. Heterodimerization of Mdm2 and Mdm4 is critical for regulating p53 activity during embryogenesis but dispensable for p53 and Mdm2 stability. Proc Natl Acad Sci USA. (2011) 108:11995–2000. 10.1073/pnas.1102241108 21730132 PMC3141986

[74] Zhang L Villa NY Rahman MM Smallwood S Shattuck D Neff C. Analysis of vaccinia virus-host protein-protein interactions: validations of yeast two-hybrid screenings. J Proteome Res. (2009) 8:4311–8. 10.1021/pr900491n 19637933 PMC2738428

[75] Thaiss CA Levy M Itav S Elinav E. Integration of innate immune signaling. Trends Immunol. (2016) 37:84–101. 10.1016/j.it.2015.12.003 26755064

[76] Sun J Wei Q Zhou Y Wang J Liu Q Xu H. A systematic analysis of FDA-approved anticancer drugs. BMC Syst Biol. (2017) 11(Suppl. 5):87. 10.1186/s12918-017-0464-7 28984210 PMC5629554

[77] Dyer MD Neff C Dufford M Rivera CG Shattuck D Bassaganya-Riera J. The human-bacterial pathogen protein interaction networks of Bacillus anthracis, Francisella tularensis, and Yersinia pestis. PLoS ONE. (2010) 5:e12089. 10.1371/journal.pone.0012089 20711500 PMC2918508

[78] Nourani E Khunjush F Durmus S. Computational approaches for prediction of pathogen-host protein-protein interactions. Front Microbiol. (2015) 6:94. 10.3389/fmicb.2015.00094 25759684 PMC4338785

[79] Brito AF Pinney JW. Protein-protein interactions in virus-host systems. Front Microbiol. (2017) 8:1557. 10.3389/fmicb.2017.01557 28861068 PMC5562681

[80] Arnold R Boonen K Sun MG Kim PM. Computational analysis of interactomes: current and future perspectives for bioinformatics approaches to model the host-pathogen interaction space. Methods. (2012) 57:508–18. 10.1016/j.ymeth.2012.06.011 22750305 PMC7128575

[81] Durmus S Cakir T Ozgur A Guthke R. A review on computational systems biology of pathogen-host interactions. Front Microbiol. (2015) 6:235. 10.3389/978-2-88919-821-4 25914674 PMC4391036

[82] Durmus Tekir S Cakir T Ulgen KO. Infection strategies of bacterial and viral pathogens through pathogen-human protein-protein interactions. Front Microbiol. (2012) 3:46. 10.3389/fmicb.2012.00046 22347880 PMC3278985

[83] Engin HB Keskin O Nussinov R Gursoy A. A strategy based on protein-protein interface motifs may help in identifying drug off-targets. J Chem Inform Model. (2012) 52:2273–86. 10.1021/ci300072q 22817115 PMC3979525

[84] Lebeis SL Kalman D. Aligning antimicrobial drug discovery with complex and redundant host-pathogen interactions. Cell Host Microbe. (2009) 5:114–22. 10.1016/j.chom.2009.01.008 19218083

[85] Anishchenko I Kundrotas PJ Vakser IA. Structural quality of unrefined models in protein docking. Proteins. (2017) 85:39–45. 10.1002/prot.25188 27756103 PMC5167671

[86] Franzosa EA Xia Y Structural models for host-pathogen protein-protein interactions: assessing coverage and bias. Pac Symp Biocomput. (2012) 2012:287–98. 10.1142/9789814366496_0028 22174284

[87] Janin J Sternberg MJ Protein flexibility, not disorder, is intrinsic to molecular recognition. F1000 Biol Rep. (2013) 5:2 10.3410/B5-02 23361309 PMC3542771

[88] Muratcioglu S Guven-Maiorov E Keskin O Gursoy A. Advances in template-based protein docking by utilizing interfaces towards completing structural interactome. Curr Opin Struct Biolo. (2015) 35:87–92. 10.1016/j.sbi.2015.10.001 26539658

[89] Gao M Skolnick J. Structural space of protein-protein interfaces is degenerate, close to complete, and highly connected. Proc Natl Acad Sci USA. (2010) 107:22517–22. 10.1073/pnas.1012820107 21149688 PMC3012513

[90] Kundrotas PJ Zhu Z Janin J Vakser IA. Templates are available to model nearly all complexes of structurally characterized proteins. Proc Natl Acad Sci USA. (2012) 109:9438–41. 10.1073/pnas.1200678109 22645367 PMC3386081

[91] Ovchinnikov S Park H Varghese N Huang PS Pavlopoulos GA Kim DE. Protein structure determination using metagenome sequence data. Science. (2017) 355:294–8. 10.1126/science.aah4043 28104891 PMC5493203

